# The tide cannot be turned without us: sex workers and the global response to HIV

**DOI:** 10.7448/IAS.16.1.18459

**Published:** 2013-08-29

**Authors:** Cheryl Overs, Bebe Loff

**Affiliations:** ^1^ Michael Kirby Centre for Public Health and Human Rights, School of Public Health and Preventative Medicine, Monash University, Melbourne, Australia

**Keywords:** sex work, new prevention technologies, HIV prevention, community participation, human rights, violence, anti‐prostitution pledge

## Abstract

Improved knowledge, better programmes and policies, effective treatment and other scientific developments have reduced levels of new HIV infections globally. Evidence shows that programmes that prevent HIV among sex workers and their clients are most successful when all aspects of vulnerability are addressed and when they are underpinned by policy that advances human rights. This is particularly important in the context of the introduction of antiretroviral‐based HIV prevention, which could have harmful consequences if not well planned. In this context, law and policy on sex work should not be limited to aiming to deliver medicine and services to sex workers in dangerous working conditions. A high‐priority aim should be to ensure that the law enables commercial sex to take place in the safest possible conditions. To achieve this, the meaningful involvement of sex workers at all levels of the response is crucial. However, although that has been recognized in theory, it has not been achieved in practice.

## Introduction

From the perspective of sex workers, the AIDS 2012 Conference was a very different conference from preceding ones. The decision to hold the conference in Washington, DC, excluded the possibility of their participation. Section 212(a)(2)(D)(i) of the US Immigration and Nationality Act states any alien who “has engaged in prostitution within 10 years of the date of application for a visa, admission, or adjustment of status” is ineligible to receive visas and ineligible to be admitted to the United States. It is notable that the mere act of engaging in prostitution, irrespective of whether a criminal act has been committed, is sufficient to be refused admission to the United States. Beyond this, according to Section 212(a)(2)(A)(i)(I), persons who have committed or attempted to commit crimes involving moral turpitude may similarly be refused admission. “Moral turpitude” is not defined by statute but is interpreted as including any base or immoral behaviour, including sexual immorality.

This comprehensive entry restriction led to the organization of a parallel conference in India that focused on sex work and HIV. The absence of sex workers from outside the United States at the AIDS 2012 Conference highlighted the need for increased attention to and investment in HIV treatment, prevention and care for sex workers, as well as law and policy reform to make sex work safer. In her speech at the conference, Secretary of State Hillary Clinton articulated for the first time the US government's commitment to HIV prevention and care for sex workers. This announcement is considered a measure of success for activism around the issue of sex work and HIV (1).

In her plenary presentation, Cheryl Overs, the founder of the Global Network of Sex Work Projects – herself previously refused admission to the United States upon landing – addressed the wide‐ranging issues facing sex workers and, in particular, highlighted the complexities inherent in the introduction of new prevention technologies to this group (2). This article is based on her presentation.

## Biomedical HIV prevention

AIDS 2012 occurred at a time of great optimism about the potential for an improved response to the HIV epidemic. Millions of people, including the most marginalized, have benefited not only from better medicines but also from better protection from discrimination, expanded harm reduction programmes and better integration of sexual and reproductive health services (3). This progress has been driven by policy and programmatic responses to factors that render any person vulnerable to HIV and its consequences: social exclusion, criminalization of sexual and other behaviours, lack of legal rights, lack of access to information about health and rights, violence, family rejection, discrimination, lack of access to health services for illnesses beyond HIV and, in the case of sex workers, dangerous working conditions.

The need for an integrated response within a human rights framework that addresses public health, economic development, and gender and social justice has been emphasized throughout the HIV epidemic. Yet limited resources mean that programme implementers have to make decisions about which commodities and services are to be given priority (4). Deciding what to prioritize has become increasingly complex with new biomedical prevention methods such as antiretroviral (ARV)‐based microbicides, pre‐exposure prophylaxis (PrEP) and treatment as prevention. These prevention methods have changed the HIV prevention landscape, with Michel Sidibé, UNAIDS Executive Director, describing them as “game‐changing” (5).

As the landscape of HIV prevention and care changes, however, there is also tense anticipation about emerging challenges. With reinvigorated emphasis on biomedical prevention, there is a risk that programmes aimed at achieving behavioural change such as community mobilization, educational programmes and advocacy will now be considered less necessary. The authors of a*Lancet* editorial have already suggested that:

Agencies such as [the] President's Emergency Plan for AIDS Relief and the Global Fund to Fight AIDS, Tuberculosis and Malaria need to reassess their prevention portfolios and consider diverting funds from programmes with poor evidence (such as behavioural change communication) to treatment for prevention. (6)

Strong reliance on biomedical technologies may substantially reduce investment in transformative behavioural change regarding HIV prevention and care. As Gary Dowsett reminds us, however, consideration of human behaviour is*the* critical underpinning and unifying factor in the application of*any* prevention technology: putting the condom on, inserting the microbicide and taking the pill all require human behaviour (7).

Furthermore, the Robert Carr doctrine, drafted by umbrella groups representing key affected populations, expresses their understanding of the constituent elements of a meaningful response to HIV. They stress, in particular, the need to address structural factors that create vulnerability:

The current response to the epidemic is underpinned by the faulty assumption that HIV is solely a public health issue. Efforts to address the epidemic are therefore centered on a narrow range of actors and strategies. In reality, the epidemic is a symptom of underlying societal inequities and injustices. … Scientific advances in HIV prevention and treatment, while welcomed and strongly encouraged, are wasted when communities shouldering a disproportionate HIV disease burden are blatantly denied access to services or cannot access them safely. (8)

## Potential problems

### Lack of research

Existing research on the likely impact of microbicides or PrEP in sex work settings has focused on assessing sex workers’ interest in using the products when they become available. Several studies have found that most sex workers are interested in using a microbicide or PrEP (9–12), and some have found that sex workers’ private partners and clients may also be willing to use them (13). What remains unclear is whether biomedical HIV prevention options are acceptable to men as an alternative to condoms or for use with condoms as dual protection. Therefore, the extent to which condoms might be abandoned for potentially less effective biomedical prevention tools (a behaviour known as “risk compensation”) remains unknown (14–16). Concerns about adherence have been noted by both sex workers and researchers (11,17).

### Cost

UNAIDS has recommended that condoms be made accessible to sex workers freely or at low cost (18). Despite this, sex workers tend to bear the cost and the responsibility for condom use. A 2011 review of literature on HIV prevention among sex workers in sub‐Saharan Africa indicates that condom cost is a reason why they may not be used (as well as client willingness to pay more for unprotected sex, or use of threats and/or brutality to obtain unprotected sex) (19). There is nothing to suggest that those who currently bear the cost (sex workers) will change with the introduction of new and more expensive prevention methods.

When it comes to medication, dose scrimping (taking reduced amounts to reduce costs) has already been identified as a risk to which sex workers may be particularly vulnerable (17). Sex workers who live in poverty are also more likely to be prey to those selling counterfeit or substandard products and medicines. Even if new HIV prevention commodities are subsidized, it is anticipated that the overall costs of the “sexual health toolkit” enabling sex workers to protect themselves against HIV, sexually transmitted infections (STIs) and pregnancy will increase. In addition, obtaining transport to a place where testing is undertaken and taking off regular time from work for this purpose constitute expenses that cannot always be met (17).

### Avoidance of health services and HIV testing

Taking an HIV test and collecting the test results can be a fraught process for sex workers, who may be fearful of discrimination, violence or the loss of livelihood contingent upon assumptions, correct or otherwise, about their HIV status. As put succinctly by this sex worker from Nepal, quoted in a UNAIDS study of the Asia Pacific,

When I visited a VCT [voluntary counselling and testing] clinic, health personnel were not polite and immediately asked me if I was a sex worker. A doctor asked me outright, “Are you HIV‐positive?” This discouraged me from going to the clinics. (18)

The conclusions of a recent study, which was conducted in Uganda among sex workers, are consistent with this comment and typical of the views expressed to the authors by sex workers globally over a period of 30 years.

The established general care health services may not cater adequately for their specific needs and accessibility may be hampered due to perceived stigma and misconceptions. The wide establishment of clinics covering and targeting high‐risk groups, offering regular screening and adequate treatment for STI, risk reduction counseling, and providing free condoms are needed to reinforce the control of the HIV epidemic in Uganda. (20)

These views are reiterated by a USAID publication, in which it is noted,

Even though we know about high levels of vulnerability to HIV and other sexually transmitted infections (STIs), sex workers nonetheless still face a host of obstacles to accessing good HIV prevention services. These obstacles include severe stigma and discrimination, which create hostile environments in some health care settings, and high numbers of violent events, which is directly linked to vulnerability to HIV. (21)

In Asia and the Pacific, country‐level data demonstrate a need to improve the uptake of HIV testing among sex workers (22). Yet sex workers – who, as is evident, avoid testing for good reason – risk contracting HIV while on PrEP and continuing with Tenofovir as monotherapy. This has been associated with the development of resistant strains of the virus (23) and compromises the individual patient's future medical outcomes (24).

### Failure to preserve confidentiality

Imparting knowledge of a positive HIV test result to a sex worker can have serious consequences.

Human Rights Watch recently published a report on the situation of sex workers in China. In a chapter entitled “Abusive public health practices against sex workers,” the report states,

Sex workers interviewed by Human Rights Watch said that they faced mistreatment by public health workers in Beijing. They described practices that violate their rights to health and privacy, including forced HIV/AIDS testing, which remains legal under Chinese law; violations of privacy and patient confidentiality; disclosure of HIV/AIDS test results to third parties; disclosure of test results to patients without provision of appropriate health services; lack of access to personal medical records; and mistreatment by health officials in charge of testing and providing health services to sex workers. These violations occur in implementation of government policies designed to curb the spread of HIV/AIDS policies that specifically identify sex workers as a “high risk” group. (25)

Elsewhere, criminal prosecution on a variety of charges may result, some of them for HIV‐specific offences and some not. Examples exist in many jurisdictions and include charges of aggravated prostitution, attempting to infect another person with an infectious disease (or HIV specifically), inflicting or attempting to inflict grievous bodily harm, reckless endangerment of life and attempted murder. This includes,*inter alia*, certain states of the United States, which have specific penalties for HIV‐positive sex workers. For example, under Title L of the Kentucky Revised Statutes, §529.090(3),

Any person who commits, offers, or agrees to commit prostitution by engaging in sexual activity in a manner likely to transmit the human immunodeficiency virus and who, prior to the commission of the crime, had tested positive for human immunodeficiency virus and knew or had been informed that he had tested positive for human immunodeficiency virus and that he could possibly communicate the disease to another person through sexual activity is guilty of a Class D felony. A person may be convicted and sentenced separately for a violation of this subsection and for the underlying crime of prostitution.

In Oklahoma, according to §21‐1031(B):

Any person who engages in an act of prostitution with knowledge that they are infected with the human immunodeficiency virus shall be guilty of a felony punishable by imprisonment in the custody of the Department of Corrections for not more than five (5) years.

Similarly, Section 39‐13‐516(a) of the Tennessee Code defines aggravated prostitution in the following way:

A person commits aggravated prostitution when, knowing that such person is infected with HIV, the person engages in sexual activity as a business or is an inmate in a house of prostitution or loiters in a public place for the purpose of being hired to engage in sexual activity.

In addition to charging sex workers with crimes of this nature, police there have posted photographs of HIV‐positive sex workers on the Internet (26,27).

The availability of home HIV tests, recently approved in the United States (28), can help sex workers to know their status and at the same time protect their privacy. Some fear, however, that home tests could be mal‐administered by police in police stations, brothels and bars in order to provide evidence with which to charge sex workers, or by brothel owners to provide clients with confidence to have the unprotected sex they seek.

### Mandatory testing and treatment

In Greece, sex workers have been forcibly subjected to HIV tests, publicly exposed and prosecuted for intentionally causing grievous bodily harm, a felony, without any evidence of risk of or actual transmission (29). Similar cases of forced testing and criminal prosecutions have emerged in Macedonia (30) and Malawi (31).

In some jurisdictions, sex work is permissible provided that conditions specified by law are observed. Documentation demonstrating that sex workers have been tested for HIV and STI is often required in order to work (28,32) (33,34). In New Zealand, documentation may not be required; however, it is an offence for commercial sex to be conducted unless reasonable precautions are taken to prevent HIV and STI transmission (35,36). Demonstrating that the sex worker has been regularly tested may be adduced as evidence that reasonable precautions have been taken. Either way, the result is the same. Mandatory testing, or its equivalent, is being required of sex workers if they are to be permitted work. Why this should be the case in legal environments where condom use in sex work is also mandated is not entirely clear.

Against this background, it is reasonable to worry that HIV testing and biomedical prevention will be similarly forced upon at least some sex workers by law or in the range of ways outlined in this section. Could it become an offence for practising sex workers to refuse to take PrEP? Could prevention services be provided only to sex workers who are willing to take PrEP (37)?

## Making sex work safe

Over‐emphasis on biomedical solutions may create a narrowing of focus from person to patient, and it may encourage a shift of education and advocacy activities from communities into clinics (38). As described in this article, sex workers are collectively the focus of stigma and discriminatory acts, and they are subject to oppressive laws and enforcement practices (35). The threat of a decline in the momentum for law reform and community‐based programmes, and an increase in biomedicalization of the epidemic, is likely to be counterproductive. Great progress has been made in the development of effective preventative technologies. However, it is not the absence of such modalities that drives the HIV epidemic but discrimination against, and exploitation and repression of, certain populations (39).

When considering how best to make sex work safe, the role of law deserves special attention. Criminalization of sex work creates inherently dangerous workplaces. This and the inability to claim ordinary legal rights and protections make sex workers vulnerable to violence and prevent them from enjoying the opportunities and protections available to other workers and citizens.

At the AIDS 2012 Conference, shocking pictures of the conditions where some sex workers operate were displayed. This included pictures of Nigerian sex workers and their clients on filthy cardboard and mattresses in bushes by the roadside in Italy (40). In the periphery, condom litter was obvious, although it is unlikely that every sexual transaction in this setting was protected. Access to ARV treatment or other healthcare among HIV‐positive women working under such conditions is likely to be low. That no person should work under such conditions is clear, and there are likely to be many views on what the best response might be. One argument is that these women should be jailed, or perhaps rescued and rehabilitated. It might be thought preferable to arrest clients. Others might argue that these women should be employed in legal, well‐managed sex businesses. The women probably have their own aspirations. Nonetheless, the most likely scenario is that there will be a police action that results in the deportation of these women or forces them to flee and work under even more dangerous conditions.

An adequate HIV response is not one that is limited to the provision of HIV prevention tools and services, even if these are delivered to sex workers where they operate. If the best that can be done for women working in bushes at the roadside, without shelter or ready access to food, is to provide them with regular HIV testing, it is obvious that HIV policy has gone deeply awry. For sex workers, an effective HIV response must address laws and policies with the aim of creating safe workplaces for sex workers, which are governed with mixes of regulations and labour and criminal law that are similar to those that apply to other workers and businesses (41). This surely offers the best chance for new prevention methods to fulfil their potential. In this vein, the Global Commission on HIV and the Law has recommended that legal measures be taken to ensure safe working conditions for sex workers, end police harassment and violence against sex workers, and prohibit mandatory HIV and STI testing of sex workers (42).

The recommendations of the Global Commission are welcome. In the short run, however, the call for decriminalization of sex work is unlikely to be widely heeded. In the interim, alternate strategies designed to produce better outcomes for sex workers are required. To date, the largest declines in STIs and HIV have been achieved in programmes that meaningfully involve sex workers (43). Practical strategies have resulted from authorities and sex workers working together. The “Ugly Mugs” programme (developed by the authors and others who were members of the Prostitutes Collective of Victoria, Australia) circulates descriptive details of people who have been violent towards sex workers. This has functioned to protect sex workers and enable police prosecution of violence against them (44). In Vietnam, arbitrary administrative detention of sex workers was ended (45). In India, the Supreme Court has required that sex workers be provided with national identity cards and other documents to enable them to claim the rights due to them as citizens (46). Human Rights Watch has described American programmes educating police not to confiscate condoms (47). Legal services assisting sex workers in court have achieved decisions that deem sex workers eligible for services and legal protections, even where sex work is illegal (39,48). Although resource allocations for such efforts are small (49), empowered sex worker communities and responsible sex business managers have effectively used what is available to resist demands for risky sex (50,51).

## Conclusions

Sex workers’ human rights claims provide important signposts demonstrating the combination of policies and programmes that can reduce HIV transmission and support the wellbeing of those living with HIV. This includes decriminalization of sex work and legal recognition of sex workers as citizens, workers and family members. These claims extend to freedom from unfair policing, forced rescue, violations of privacy, violence and enforced medical procedures. As regards new biomedical prevention methods, for HIV prevention in particular, if they are to live up to their potential, they must be introduced in tandem with programs that support the development of safe and respectful workplaces and communities. Evidence‐based guidance is required to ensure that new technologies are used correctly, and that potential risks to sex workers, such as those discussed in this article, are identified and reduced.

Elena Reynaga, coordinator of the Latin American Sex Workers Network, made similar points in her 2008 plenary speech at the Mexico AIDS conference. She stressed the importance of meaningful involvement of sex workers at all levels of the response to AIDS. She challenged governments and donors to provide resources directly to sex workers’ organizations to build and, where possible, implement research, advocacy campaigns and interventions. This challenge has not been met. It is our hope that by the time of the International AIDS Conference in Melbourne in 2014, some advances towards meeting this call will be made by presenting better evidence of results from existing interventions and policies as well as clearer strategies for ensuring that biomedical HIV prevention benefits sex workers, causes no harm and reduces HIV transmission during commercial sex.

## Competing interests

None declared.

## Authors' contributions

This article is based on the plenary presentation by CO at the AIDS 2012 Conference. CO conceptualized the manuscript and wrote the first draft. BL added further thoughts and contributed to the writing. Both authors have read and approved the final manuscript.

## Acknowledgements

The authors would like to acknowledge the invaluable contributions of Cassandra Van, Michael Williams, Anatoli Shapiro, Matthew Greenall and The Roddick Foundation UK.
